# Gold Crown

**Published:** 1903-04-15

**Authors:** H. E. Jenkins


### GOLD CROWN.

BY H. E. JENKINS.

The unsightly appearance of a gold crown makes it desirable for the back of the mouth, and the farther back the better. Make a seamless crown by carving a tooth model in beeswax. A plaster cast of the adjoining and opposing teeth is absolutely necessary in order to get a perfect articulation, size and contour; then embed wax model in asbestos and plaster in halves, separate, heat them and pour full of type metal which gives a tooth the exact duplicate of the one made in wax. Set this on a base and put a metal ring over it, pour full of any of the low fusible metals, separate and put in seamless capsule which has been drawn to the size of the circumference of the neck of metal tooth; place back in ring, fill with very fine shot and swage until formed. This gives a crown that has a perfect articulation, no matter how irregular, and one that is contoured on the proximal surface; thus preventing any wedging of food, which is a very common trouble with crowns.

Use 22k gold and always reinforce the cusps with 18 or 20k solder.

Make it an invariable rule not to put a crown on a live tooth, for in such a case trimming causes the patint excruciating pain if the tooth is trimmed so the metal crown will not be any larger than the natural one. So much trimming often kills the pulp, so have abandoned it and prefer devitalizing and filling root, cutting off the tooth to the gum line when it can be trimmed to suit. This may seem like a long and tedious way to make a crown, but after having made several of them you will find that it takes no more time than making a band and soldering on the cap. The above method requires about an hour and a quarter and most of this time is lost in waiting for plaster to harden at the start.

All bridges back of the first bicuspid should be entirely of gold. Make dummies by the same method as for crowns; reinforcing them with solder, cutting off the inner portion to angle desired, cover with plate of gold and solder, which makes the dummies hollow and sufficiently strong.

For the front of the mouth use porcelain carved or facings. The facings are most satisfactory for bridges. If the occlusion is close use a gold backing, if not, porcelain, which is strengthened by a square bar of heavy irido-platinum extending from one abutment to the other, to which the pins are soldered with a 25 percent platinum solder.

### CARVED PORCELAIN CROWNS.

It requires patience and perseverance to master the art of making carved porcelain crowns, but when properly made we have the nearest approach to nature from a hygienic and artistic standpoint.

The post and band are the same as for a Richmond crown except platinum is used in place of gold and the post extends into body of crown to which the porcelain attaches itself, thereby giving strength. I use the high-fusing bodies.

When carving the tooth it is essential for the operator to have artistic tendencies and be able to model, otherwise the beauty of the carved crown is lost. It takes an eye for color as well as some experience to learn to color properly. Facings may be used in this kind of work and backs with porcelain.

Use an electric furnace and depend on the eye as a guide rather than time in baking. Remember to allow for shrinkage about 1-6. The porcelain will fuse band and the post that runs through the cap gives strength to the crown and makes a water-tight joint that will not discolor as does a Richmond crown.

They can be made in any odd shape, and are practically indestructible, match the natural teeth, and are most satisfactory crowns.

